# Expression optimization of a cell membrane-penetrating human papillomavirus type 16 therapeutic vaccine candidate in *Nicotiana benthamiana*

**DOI:** 10.1371/journal.pone.0183177

**Published:** 2017-08-11

**Authors:** Romana J. R. Yanez, Renate Lamprecht, Milaid Granadillo, Brandon Weber, Isis Torrens, Edward P. Rybicki, Inga I. Hitzeroth

**Affiliations:** 1 Biopharming Research Unit, Department of Molecular and Cell Biology, University of Cape Town, Rondebosch, Cape Town, South Africa; 2 Center for Genetic Engineering and Biotechnology, Cubanacan, Playa, Havana, Cuba; 3 Structural Biology Research Unit, Division of Medical Biochemistry, Department of Clinical Laboratory Sciences, University of Cape Town, Observatory, Cape Town, South Africa; 4 Institute of Infectious Disease and Molecular Medicine, University of Cape Town, Observatory, Cape Town, South Africa; Università della Calabria, ITALY

## Abstract

High-risk human papillomaviruses (hr-HPVs) cause cervical cancer, the fourth most common cancer in women worldwide. A HPV-16 candidate therapeutic vaccine, LALF_32-51_-E7, was developed by fusing a modified E7 protein to a bacterial cell-penetrating peptide (LALF): this elicited both tumour protection and regression in pre-clinical immunization studies. In the current study, we investigated the potential for producing LALF_32-51_-E7 in a plant expression system by evaluating the effect of subcellular localization and usage of different expression vectors and gene silencing suppressors. The highest expression levels of LALF_32-51_-E7 were obtained by using a self-replicating plant expression vector and chloroplast targeting, which increased its accumulation by 27-fold compared to cytoplasmic localization. The production and extraction of LALF_32-51_-E7 was scaled-up and purification optimized by affinity chromatography. If further developed, this platform could potentially allow for the production of a more affordable therapeutic vaccine for HPV-16. This would be extremely relevant in the context of developing countries, where cervical cancer and other HPV-related malignancies are most prevalent, and where the population have limited or no access to preventative vaccines due to their typical high costs.

## Introduction

Cancer is a leading cause of death globally, and high-risk human papillomaviruses (hr-HPVs) have been linked to numerous genital and oropharyngeal malignancies. Of these, the most important is cervical cancer, which is the fourth most common cancer in women worldwide [1,2]. There are approximately 530,000 new cases of cervical cancer and 270,000 deaths per year, of which, more than 85% occur in developing countries [3,4]. Essentially all (99.7%) cervical cancer cases are caused by persistent infections with hr-HPVs. The most prevalent types worldwide are HPV-16 and -18, accounting for more than 70% of cases [5–7].

Currently, there are three commercially available vaccines against HPVs: these are Cervarix^®^ (GlaxoSmithKline Inc.), Gardasil^®^ and Gardasil^®^ 9 (Merck & Co., Inc.). All exploit the fact that the HPV major capsid protein L1 can form virus-like particles (VLPs) when expressed alone in a variety of cell types, which are morphologically and antigenically very similar to native virions [8]. These vaccines prevent HPV infections caused by the targeted types by eliciting neutralizing antibodies [9–11]. However, they are not effective at eliminating pre-existing infections [12–14]. Therapeutic HPV vaccines that can eliminate established infections are therefore still needed—and these need to be affordable, given the burden of HPV disease is mainly in developing countries [4,12].

The early-expressed E7 oncoprotein of hr-HPVs is essential for the onset and maintenance of the transformed cell phenotype. It is expressed constitutively and at high levels in infected cells, and therefore represents an ideal target for therapeutic vaccine candidates [5,15,16]. Granadillo *et al*. (2011) [17] developed a HPV-16 E7-based therapeutic vaccine produced in an *Escherichia coli* expression system. This consisted of the E7 oncoprotein fused to a peptide derived from the *Limulus polyphemus* anti-lipopolysaccharide factor (LALF_31-52_). This is a small cationic and amphipathic peptide that can penetrate cell membranes and that also has immunomodulatory properties [18]. The vaccine had both protective and therapeutic qualities in a mouse model: mice were protected against tumour formation after challenge with the E7-expressing TC-1 cell line, and established tumours were significantly reduced following vaccination, with significant tumour-specific cell-mediated immune responses being elicited. These results confirmed the potentiating effect of the cell-penetrating peptide, and showed that LALF_32-51_-E7 has promise as a HPV therapeutic vaccine. However, production in bacteria was problematic as yields were low and the product was highly insoluble.

Thus, in the current study we investigated producing LALF_32-51_-E7 transiently in a plant expression system, as an alternative to bacteria. Plant expression systems offer significantly lower upstream production costs compared to bacterial, yeast or mammalian cell systems [19–25]. Additionally, plants are not susceptible to animal pathogen contamination like mammalian or insect cells, they do not produce endotoxins like bacterial cultures, and they can also post-translationally modify proteins similarly to mammalian cells. These properties make plant production of biologics potentially more efficient, safer and more cost-effective than conventional expression systems. Furthermore, plant production is highly scalable, from laboratory benchtops through to agricultural scale in fields, greenhouses or in vertical farming units [22–25]. Plants have also been shown to produce biologically functional pharmaceuticals, including products presently in use in humans such as glucocerebrocidase [22,26–29].

In this study, we aimed at optimizing the expression levels of LALF_32-51_-E7 in plants by use of a self-replicating expression vector, different subcellular localizations and the use of gene silencing suppressors. The self-replicating plant expression vector pRIC3.0 is based on the mild strain of the bean yellow dwarf mastrevirus (BeYDV), and its use in amplifying gene expression has been amply described [30,31]. Subcellular localization by targeting proteins to the chloroplasts, the endoplasmic reticulum (ER) or the apoplast can have a dramatic effect on accumulation levels of recombinant proteins [32–34], possibly because proteins may be less prone to degradation in these locations than in the cytoplasm [34–37]. Given promising results in preliminary investigations, we therefore investigated the viability of producing LALF_32-51_-E7 by targeting it to the chloroplasts of *N*. *benthamiana* leaves.

Posttranscriptional gene silencing (PTGS) often negatively affects the expression of recombinant proteins in plants. Accordingly, we used two well-known silencing suppressors, the tomato spotted wilt virus NSs protein [38] and the tomato bushy stunt virus P19 protein [39], to investigate their potential for increasing the accumulation levels of LALF_32-51_-E7 in *N*. *benthamiana*.

While scaling-up the expression of plant-produced recombinant protein is not affected by challenges faced by other systems such as having to build complex and expensive bioreactors [24], the downstream processing costs are similar to those of other expression systems, and account for the bulk of production costs. It is therefore important to optimize large scale expression, extraction and especially purification [24,40,41]. When expressed in *E*. *coli*, LALF_32-51_-E7 could be purified using a single step of Ni^2+^ affinity chromatography [17,42]. Accordingly, in this study, we investigated a similar purification step after scaling up the production of LALF_32-51_-E7 in *N*. *benthamiana* plants.

## Results

### Comparing the accumulation of LALF_32-51_-E7 in the cytoplasm versus the chloroplasts

**Fig 1:**
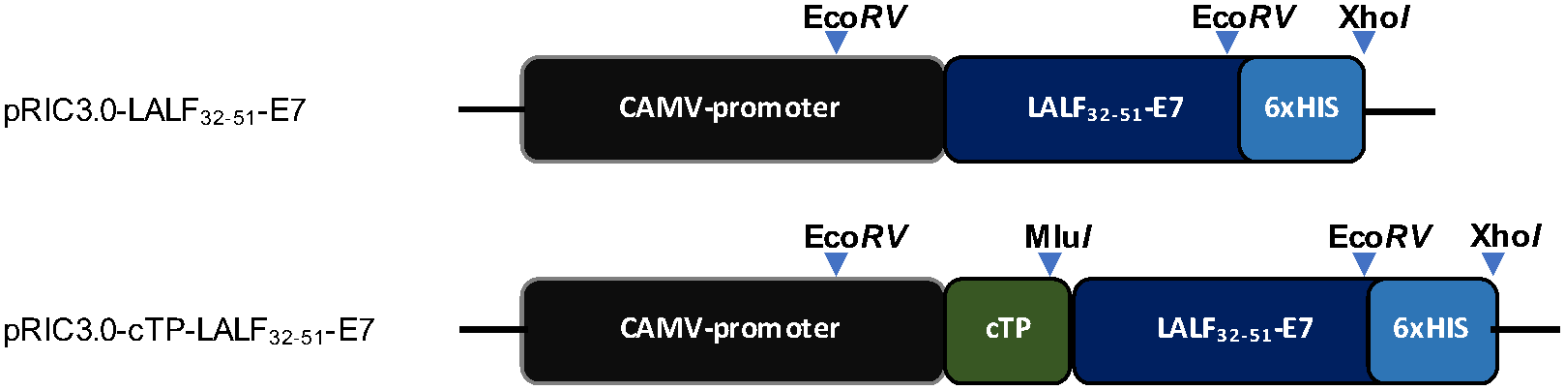
Construct generation for the expression of LALF_32-51_-E7 in *N*. *benthamiana*. The LALF_32-51_-E7 sequence was plant codon-optimized and inserted into the self-replicating plant expression vector pRIC3.0, with and without a chloroplast targeting peptide (cTP) signal. Light-blue arrows, restriction enzyme sites. CAMV-promoter, the cauliflower mosaic virus 35S constitutive promoter. 6xHIS, hexa-histidine tag. Not drawn to scale.

Previously the first cysteine of HPV-16 E7 sequence was modified in order to disrupt the binding site of E7 to retinobastoma protein to eliminate its oncogenic ability [17]. The LALF_32-51_-E7 fusion protein sequence was codon-optimized for expression in *N*. *benthamiana* and inserted into the self-replicating plant expression vector pRIC3.0 with and without the chloroplast transit peptide (cTP). cTP is derived from the potato rbcS1 gene of the ribulose-1,5-bisphosphate carboxylase/oxygenase (RuBisCO) small subunit and targets the protein to the chloroplast. Electrocompetent *A*. *tumefaciens* were transformed with the final constructs shown in Fig 1.

**Fig 2:**
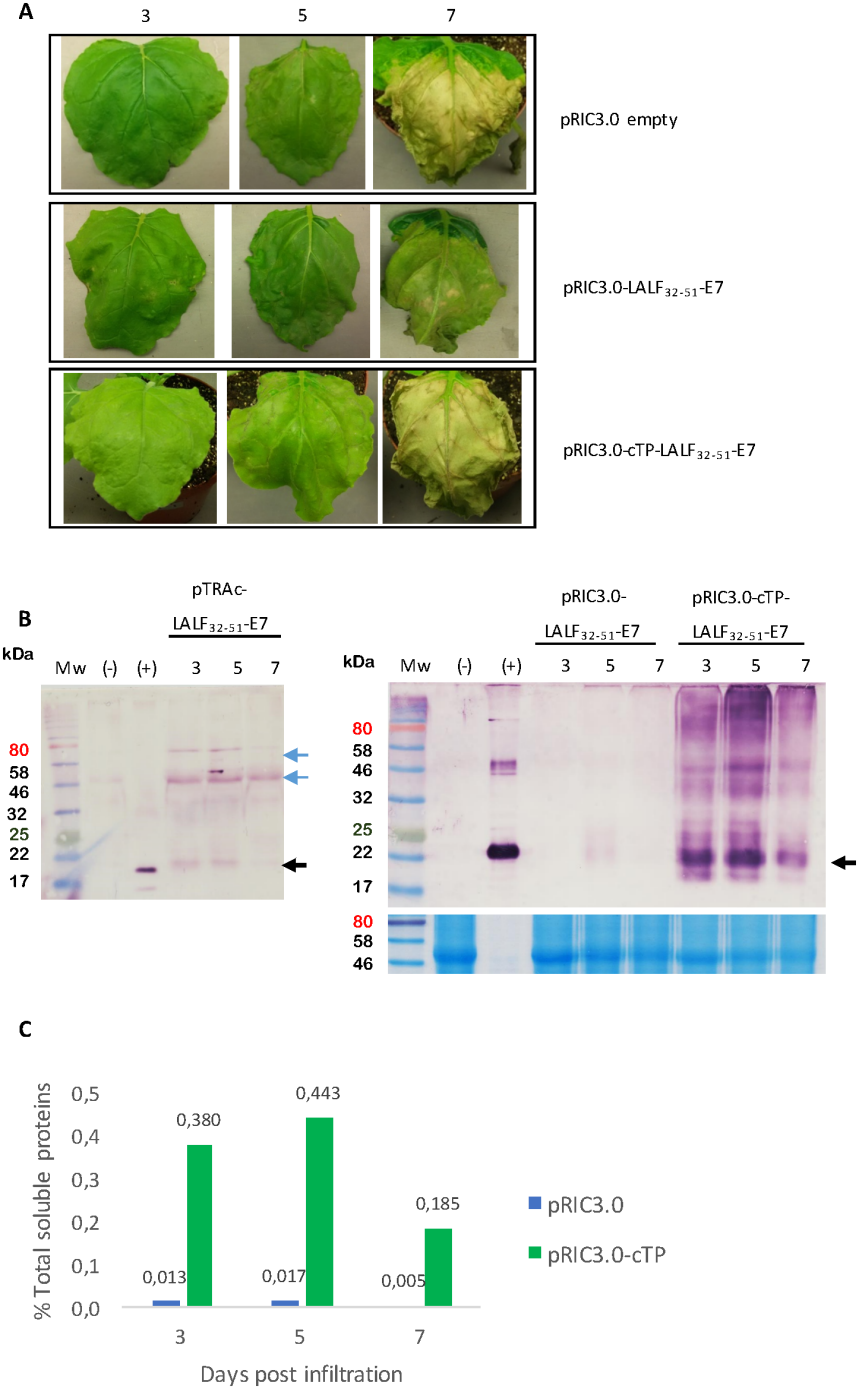
Comparing the effects of expression vector and subcellular localization on the expression of LALF_32-51_-E7. **(A),** Leaf physiology monitored over time after vacuum infiltration with pRIC3.0-LFLF_32-51_-E7, pRIC3.0-cTP-LFLF_32-51_-E7 and pRIC3.0 empty. All cultures used were set to an OD_600_ of 1.0. Shown are representative leaves on 3, 5 and 7 dpi of three independent repeats. **(B)** The effect of expression vector on LALF_32-51_-E7 accumulation. Left panel, equal volume western blots of LALF_32-51_-E7 expression from pTRAc using an antibody dilution of 1:1,000. (+), purified *E*. *coli*-derived LALF_32-51_-E7. (-), pRIC3.0 empty vector crude extract. Black arrow indicates the expected position of LALF_32-51_-E7, ≈22 kDa. Blue arrows show higher molecular weight aggregates. Right panel, analysis of 50 μg of TSP of pRIC3.0-LALF_32-51_-E7 and pRIC3.0-cTP-LALF_32-51_-E7 crude extracts. Western blot using an antibody dilution of 1:5,000 (top) and corresponding AquaStained SDS-PAGE gels showing a native protein of approximately 50 kDa as an internal control for equal TSP loading (bottom). (-), pRIC3.0 empty vector crude extract. (+), *E*. *coli*-derived LALF_32-51_-E7 inclusion bodies. Arrow indicates the position of LALF_32-51_-E7, ≈22 kDa. Mw, molecular weight marker. TSP, total soluble proteins. **(C)** The effect of subcellular localization on LALF_32-51_-E7 accumulation determined by the %TSP.

A pilot small-scale syringe-infiltration was done to determine the optimal optical density (OD_600_) and expression profile of LALF_32-51_-E7 in pRIC3.0 and pRIC3.0-cTP (S1 Fig). To compare the accumulation of LALF_32-51_-E7 in the cytoplasm versus the chloroplasts, leaves were vacuum-infiltrated with pRIC3.0-LALF_32-51_-E7 and pRIC3.0-cTP-LALF_32-51_-E7, using the overlapping optimum OD_600_ of 1.0. The constructs pTRAc-LALF_32-51_-E7 and pRIC3.0 empty were used as controls. Infiltrated leaves were monitored over 7 days. On 5 days post infiltration (dpi) signs of damage were seen, which became severe by 7 dpi, including brittle leaves and chlorosis (Fig 2A).

Equal volume western blots showed how LALF_32-51_-E7 was almost undetectable when expressed from pTRAc, even when detecting with concentrated antibodies (Fig 2B, left panel). However, large molecular weight aggregates were detected in these samples at approximately 46 and 80 kDa. Equal total soluble protein (TSP) western blots of pRIC3.0-LALF_32-51_-E7 and pRIC3.0-cTP-LALF_32-51_-E7 were used to analyse the accumulation of LALF_32-51_-E7 in the cytoplasm versus the chloroplasts (Fig 2B, right panel). The size of the chloroplast targeted protein was the same as the cytoplasmic one indicating that the cTP is cleaved off when it is transported into the chloroplast. When expressed in pRIC3.0, the accumulation of LALF_32-51_-E7 peaked on 5 dpi and was undetectable on 3 and 7 dpi. As determined by western blot densitometry, it accounted for up to 0.017% TSP in the extracts analysed (Fig 2C). On the other hand, the accumulation of LALF_32-51_-E7 when expressed in pRIC3.0-cTP fluctuated less than in pRIC3.0, peaking on 3 and 5 dpi. This accounted for up to 0.56 (±0.08)% TSP however on average this was 0.44% TSP. The accumulation of LALF_32-51_-E7 in the chloroplasts was 26.8-fold higher than the accumulation in the cytoplasm when considering the average %TSP on 3 and 5 dpi. No LALF_32-51_-E7 was detected in pRIC3.0 empty vector extracts.

### Investigating the effect of silencing suppressors on the expression of LALF_32-51_-E7

**Fig 3:**
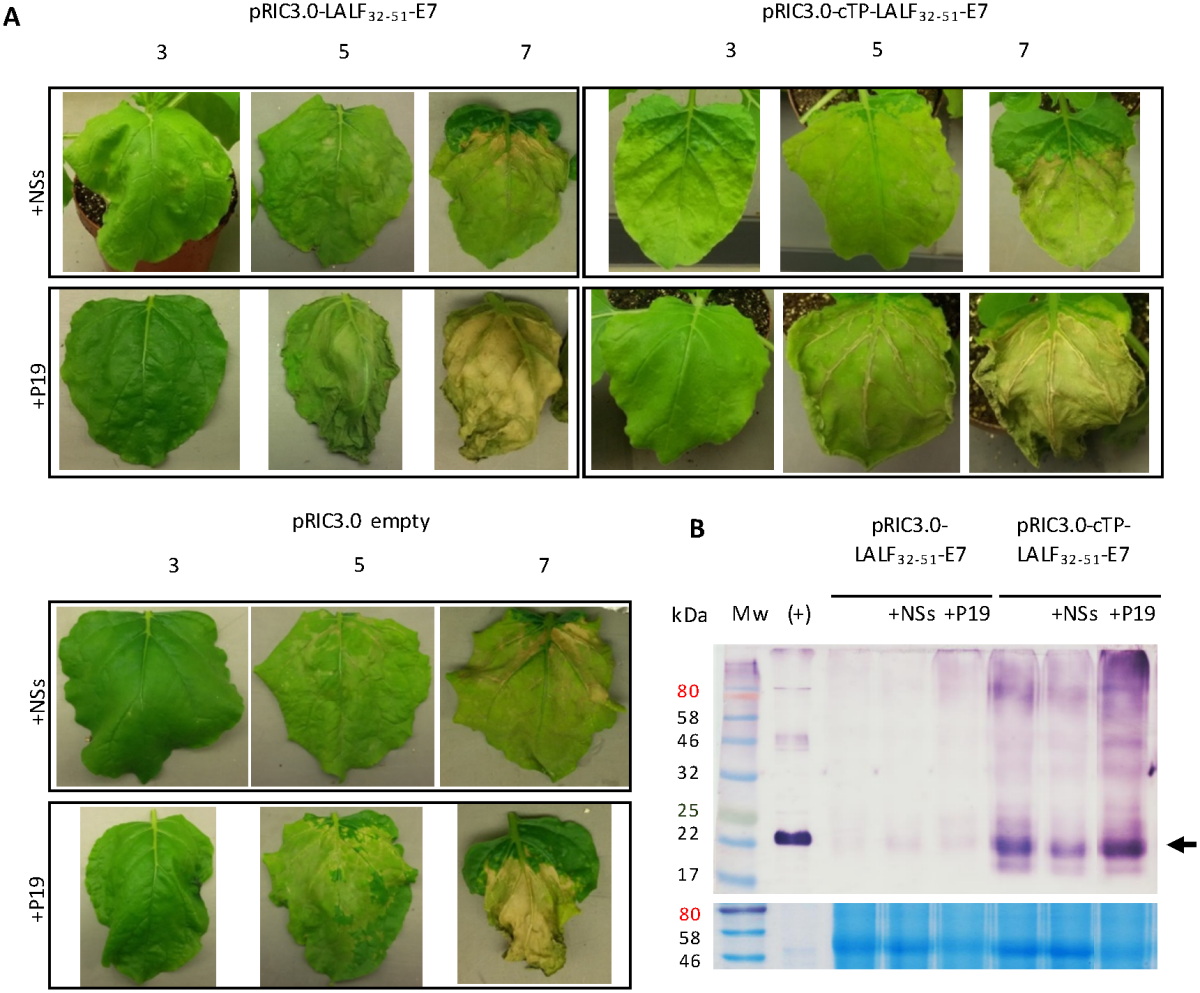
The effects of silencing suppressors on the expression of LALF_32-51_-E7. **(A)** Leaf physiology monitored over 7 days after vacuum co-infiltration of silencing suppressors and pRIC3.0-LALF _32-51_-E7 or pRIC3.0-cTP-LALF _32-51_-E7. All cultures used were set to an OD_600_ of 1.0. Shown are representative leaves on 3, 5 and 7 dpi. pRIC3.0 empty vector was used as a negative control. **(B)** Analysis of 50 μg of TSP crude extracts of leaves vacuum-infiltrated with or without a silencing suppressor on the best expression dpi. Top panel, western blot using an antibody dilution of 1:5,000. Bottom panel, corresponding AquaStained SDS-PAGE gel showing a native protein of approximately 50 kDa as an internal control for equal TSP loading. (+), *E*. *coli*-derived LALF _32-51_-E7 inclusion bodies. Arrow indicates the position of LALF _32-51_-E7 ≈22 kDa. Shown here are representative images of two independent repeats. Mw, molecular weight marker. TSP, total soluble protein.

To determine the effect of silencing suppressors on the expression of LALF_32-51_-E7 in *N*. *benthamiana*, leaves were co-infiltrated with pRIC3.0-LALF_32-51_-E7 or pRIC3.0-cTP-LALF_32-51_-E7 and P19 or NSs. The pRIC3.0 empty vector was used as a negative control. The plants were monitored over 7 days and we observed that signs of necrosis were exacerbated by the co-infiltration with P19, and seemed to be attenuated by co-infiltration with NSs (Fig 3A).

Western blot analysis of leaf extracts from each construct and silencing suppressor combination showed that LALF_32-51_-E7 in pRIC3.0 peaked on 5 dpi, with or without silencing suppressor, which was consistent with previous experiments. The best day for pRIC3.0-cTP-LALF_32-51_-E7 was 3 dpi, except when in the presence of P19, in which case it was 5 dpi. No LALF_32-51_-E7 was detected in pRIC3.0 empty vector leaf extracts. Based on the above results, TSP content of the leaf extracts from the best expression day of each combination was determined. Equal TSP western blots showed that the expression of LALF_32-51_-E7 in pRIC3.0 did not seem to change in the presence of either silencing suppressor (Fig 3B). It was not possible to accurately quantify these samples due to their very low level of accumulation. The expression of LALF_32-51_-E7 in pRIC3.0-cTP only increased in the presence of P19, on average by 1.3-fold, while it seemed to decrease by on average 0.92-fold in the presence of NSs.

### Optimization of LALF_32-51_-E7 extraction

**Fig 4:**
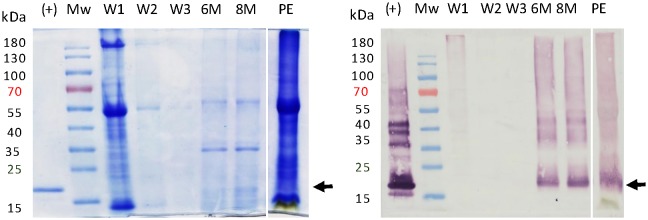
Enhanced LALF_32-51_-E7 extraction strategy. Leaf material expressing LALF_32-51_-E7 was ground-up with liquid nitrogen and washed 3 times with PBS (W1-3). LALF_32-51_-E7 was solubilized from the leaf material with extraction buffer containing 6 M or 8 M urea (6M and 8M, respectively). The final extracts are compared to a crude extract sample prepared using the previous extraction strategy (PE). (+), purified *E*. *coli*-produced LALF_32-51_-E7. Left panel, Coomassie-stained SDS-PAGE gel. Right panel, western blot using an antibody dilution 1:5,000. Arrows indicate the position of LALF_32-51_-E7 ≈ 22 kDa. Mw, molecular weight marker.

Once the parameters for optimal expression and accumulation of LALF_32-51_-E7 in *N*. *benthamiana* leaves were finalised, we investigated scaling up its production and development of an extraction and purification strategy. An approach for the extraction of LALF_32-51_-E7 was developed which was analogous to the extraction of the *E*. *coli*-produced counterpart, described in [42]. Homogenised leaf material was washed with phosphate buffer saline (PBS) to remove the majority of the plant host soluble proteins, while the highly hydrophobic LALF_32-51_-E7 stayed in the insoluble fraction. Thereafter, LALF_32-51_-E7 was solubilized from the plant material with a urea extraction buffer. This extraction strategy worked as expected (Fig 4): no LALF_32-51_-E7 was lost during the PBS washes, while the bulk of host contaminant proteins were removed (See Fig 4, lanes 6M, 8M and PE) and the final crude extract was clear and non-viscous. LALF_32-51_-E7 was better extracted with a concentration of 8 M urea. Furthermore, the addition of Triton X-100 seemed to increase the extraction efficiency of LALF_32-51_-E7.

### Purification of LALF_32-51_-E7 by Ni^2+^ affinity chromatography

**Fig 5:**
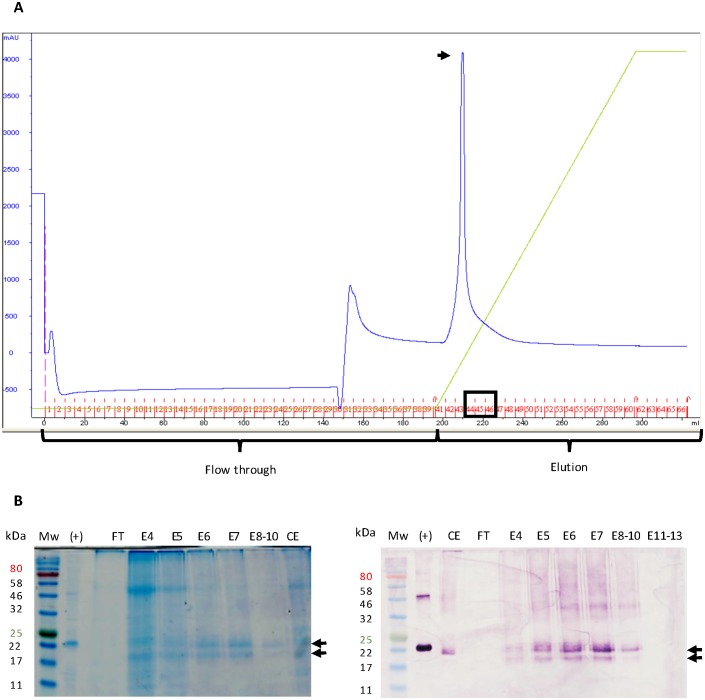
Purification of LALF_32-51_-E7 using Ni^2+^ column chromatography. **(A)**, LALF_32-51_-E7 purification chromatogram. Blue curve, A_280_ representing protein levels. Light-green curve, elution buffer gradient. Arrow shows the elution peak corresponding to elution fractions 1–7. Black box, elution fractions 5–7 corresponding to LALF_32-51_-E7. **(B)**, analysis of relevant fractions by AquaStained SDS-PAGE gel (left panel) and anti-E7 polyclonal antibody western blot using dilution of 1:5,000 (right panel). (+), *E*. *coli*-LALF_32-51_-E7 inclusion bodies. CE, LALF_32-51_-E7 crude extract from 50 g fresh leaf weight. FT, flow through. E4-E13, elution fractions 4–13. Arrows point at the doublet bands representing LALF_32-51_-E7 ≈ 20 and 22 kDa. Mw, molecular weight marker.

His-tagged LALF_32-51_-E7 was purified by Ni^2+^ affinity chromatography on an ÄKTA Explorer^™^ (GE Healthcare Life Sciences) system. Approximately 50 g fresh leaf weight (FLW) of starting material were used. The purification process was repeated several times, testing different conditions. When extracting LALF_32-51_-E7 with large volumes (5–6 times v/w) of extraction buffer, the purified LALF_32-51_-E7 concentration was very low, as indicated by A_280_ values of 1,100 to 1,300 milli absorption units (mAU). However, when the extraction volume was between 2–3 v/w, more concentrated LALF_32-51_-E7 was obtained and within fewer elution fractions, resulting in an A_280_ value of more than 4,000 mAU. The purification process was optimised by the elimination of the low pH wash step between and after sample binding and the absence of imidazole in the crude extract and equilibration buffer (Fig 5A).

The Ni^2+^ chromatography elution fractions were initially analysed by immunodot-blots to determine the LALF_32-51_-E7-containing fractions. The identified fractions did not correspond to the elution peak, instead they corresponded to adjacent later fractions. This suggested that another protein or proteins eluted with LALF_32-51_-E7. The AquaStained gel of these fractions showed a high molecular weight band between 46 and 58 kDa (Fig 5B). Furthermore, LALF_32-51_-E7 appeared as a double band in the stained gels and in the western blots.

**Fig 6:**
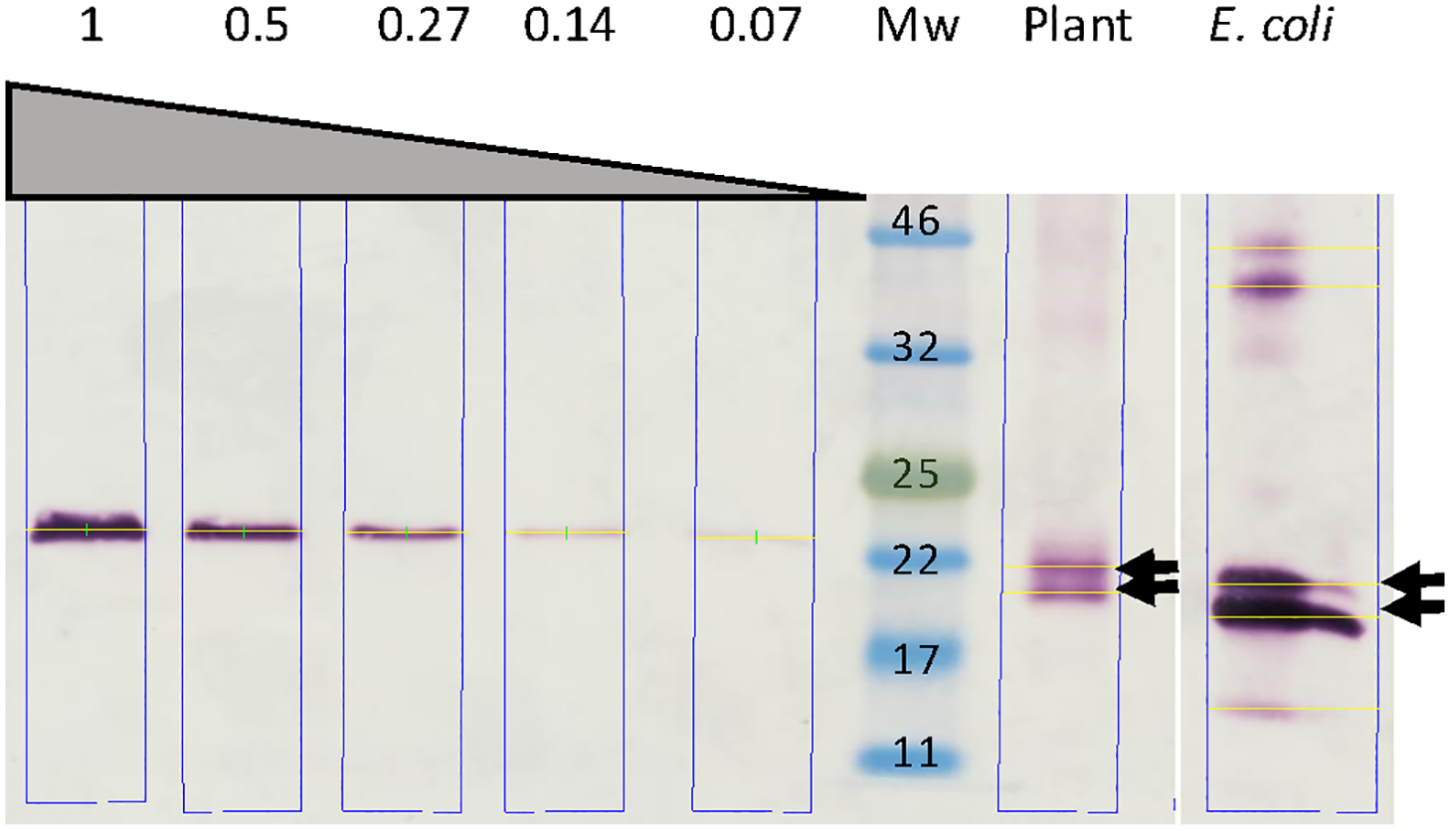
Quantification of partially purified LALF_32-51_-E7. Relevant fractions obtained during the purification of LALF_32-51_-E7 by Ni^2+^ affinity chromatography were pooled and dialyzed against Tris buffer. The dialyzed LALF_32-51_-E7 was then quantified by western blot densitometry using *E*. *coli*-produced LALF_32-51_-E7 inclusion bodies as standard—grey triangle with loaded amounts in μg shown above. Plant, partially purified and dialyzed plant-produced LALF_32-51_-E7. *E*. *coli*, purified *E*. *coil*-produced LALF_32-51_-E7. Arrows point at the LALF_32-51_-E7 doublet ≈ 20 and 22 kDa. Mw, molecular weight marker.

LALF_32-51_-E7-containing chromatography fractions were pooled and dialysed against Tris buffer and LALF_32-51_-E7 was quantified by western blot densitometry, with *E*. *coli*-produced LALF_32-51_-E7 inclusion bodies as a standard (Fig 6). The yield of the purified plant-produced LALF_32-51_-E7 was 15 mg/kg FLW at a concentration of 0.0231 mg/ml. The expression of LALF_32-51_-E7 from pRIC3.0-cTP on 3 dpi was extrapolated to be on average 50 mg/kg FLW. Therefore, 30% of LALF_32-51_-E7 was recovered during the purification process.

## Discussion

The fusion protein LALF_32-51_-E7 is a potential therapeutic vaccine candidate for HPV-16. It was previously expressed in *E*. *coli* and was successful in eliciting tumour protection and tumour regression in a mouse animal model [17,42]. However, it would be potentially more economical to use plant expression systems to produce this candidate vaccine, given that the expression is reasonably high [19,20].

While preliminary experiments proved that LALF_32-51_-E7 could be expressed in *N*. *benthamiana* leaves, expression levels were very low. Accordingly, the aim of this study was to increase and optimize its expression levels by using a replicating plant expression vector and silencing suppressors. Expression of LALF_32-51_-E7 was successful using both pRIC3.0 and pRIC3.0-cTP vectors. This agreed with the results of Regnard et al. (2010) [30] and others [43–46] in that significantly increasing gene copy number can lead to an increase in the expression of recombinant proteins. It is interesting that this occurs despite the vector inducing distinct infection-like symptoms in the plants, even when no insert was present. Other studies have reported that the Rep proteins of similar geminiviruses can induce hypersensitive responses in plants [47]. However, the protein expression levels were not negatively affected by these symptoms, even on 7 dpi, compared to the expression obtained from non-replicating vectors. It was noted that LALF_32-51_-E7 migrated at ≈ 22 kDa in western blots, while its predicted size is ≈ 15 kDa. This was consistent with observations by Granadillo et al. (2011) [17]. The net negative charge of E7 is known to influence the migration of this protein in SDS-PAGE gels, making it appear larger [48]. This has also been observed with other E7-based fusion proteins, like 16E7SH [28]. Targeting LALF_32-51_-E7 to the chloroplasts resulted in the highest accumulation levels obtained, representing an increase in %TSP of 26.8-fold, compared to LALF_32-51_-E7 accumulation levels in the cytoplasm. This agrees with the hypothesis that proteins sequestered in cell compartments are less prone to degradation. It is thought that protease concentrations are lower in these compartments than in the cytoplasm [19,35,37]. The fact that targeting LALF_32-51_-E7 to the chloroplasts dramatically increased its accumulation levels compared to localizing it in the cytoplasm, suggest that subcellular localization played a greater role in the accumulation of LALF_32-51_-E7 than the increase in gene copy number provided by the self-replicating vector. This agrees with the findings of Regnard et al. (2010) [30], in that the increase in gene copy number was not proportional to the fold increase in protein expression. We were able to prove that the chloroplast transit peptide did target the protein to the chloroplast by fusing the LALF_32-51_-E7 to enhanced GFP and determining localization by confocal laser scanning microscopy and determining that it formed protein body-like structures which clearly localized with the chloroplast [49].

Previous work with the protein body-forming E7 fusion protein ZERA-16E7SH expressed using the non-replicating, non-targeting expression vector pTRAc showed that the accumulation of this protein was enhanced in the presence of the NSs protein [28]. Accordingly, *N*. *benthamiana* leaves were co-infiltrated with pRIC3.0-LALF_32-51_-E7 or pRIC3.0-cTP-LALF_32-51_-E7 and the NSs or P19 silencing suppressors. It was shown that co-infiltration with silencing suppressors did not have a large impact on the expression of LALF_32-51_-E7, as the increase was only 1.3-fold. This can be considered insignificant when compared to the 26.8-fold increase seen when targeting to the chloroplast versus cytoplasmic localization. The only instances where silencing suppressors did seem to influence the expression of LALF_32-51_-E7 were with the 7 dpi samples. This is not surprising as PTGS takes place in later stages of infection. However, the expression levels at 7 dpi in the presence of silencing suppressors were lower than on days 3 and 5 with or without silencing suppressors. The replication of geminivirus-based vectors is rapid and high expression levels are often obtained before the onset of PTGS [50]. Thus, amplification of gene copy number by the self-replicating vectors can have a greater impact on expression levels than that of silencing suppressors. We therefore concluded that the combined effects of increased gene copy number and the subcellular localization of LALF_32-51_-E7 had the major impacts on its accumulation levels, compared to the effects of either of the silencing suppressors. This is interesting, as the two proteins act at different levels in PTGS: NSs is a RNA silencing suppressor from the *Tomato spotted wilt virus* (TSWW) and it inhibits the initial stages of the silencing pathway by possible interaction with RdRP [38], while P19 from *Tomato bushy stunt virus* binds to siRNAs preventing siRNA incorporation into RISC [38,51]. NSs only suppresses sense transgene-induced PTGS but does not suppress inverted repeat transgene-induced PTGS; however, p19 has an effect on both arms of silencing as it acts at the siRNA level, which may be the reason of the increased signs of necrosis observed in the plants infiltrated with p19 in this study [40].

In the scale up investigation, we opted to express LALF_32-51_-E7 targeted to the chloroplasts in the absence of silencing suppressors, as infiltrating with just one bacterial strain would simplify the scaling-up process and would be more economical in a possible future industrial set-up. For consistency, pRIC3.0-cTP-LALF_32-51_-E7 *A*. *tumefaciens* cultures at an OD_600_ of 1.0 were used during the large-scale production of LALF_32-51_-E7. However, this could be reduced to 0.5 as it was equally effective.

The extraction strategy we applied was analogous to the extract preparation used for *E*. *coli*-produced LALF_32-51_-E7 [42]. Most soluble host proteins were successfully removed by washing the homogenised plant material with PBS before solubilising LALF_32-51_-E7 with urea. This protocol represented a more efficient way of extracting LALF_32-51_-E7 from plant material, compared to directly solubilizing it with a urea buffer without previous PBS washes.

The purification of LALF_32-51_-E7 by means of metal-ion affinity chromatography was complicated by the fact that it eluted at a low imidazole concentration, suggesting that it was weakly bound to the Ni^2+^ resin. A small portion of LALF_32-51_-E7 co-eluted with most of the contaminant proteins in early elution fractions, while it was more strongly detected in later elution fractions. The contaminant proteins were not found in the flow-through, as seen in the chromatogram and stained gel in Fig 5. The apparent interaction of LALF_32-51_-E7 and host proteins was not eliminated by the denaturing conditions used in the process, nor by the addition of the detergent Triton X-100, suggesting this association was strong. Alternatively, the early-eluting portion could be high molecular weight aggregates of LALF_32-51_-E7 not recognized by the antibodies used. The purified plant-produced LALF_32-51_-E7 appeared as a double band in SDS-PAG electropherograms: this was also seen for the *E*. *coli*-produced LALF_32-51_-E7. Similar doublet formation for HPV-16 E7 was observed by Demurtas et al. (2013) [52], who proposed these were phosphorylated forms of the protein. The HPV-16 E7 protein is known to be phosphorylated at serine residues [53].

Overall, the use of Ni^2+^ affinity chromatography was not much more than a polishing step in the purification of LALF_32-51_-E7. The protein in fact appeared to be purified to the greatest extent by the pre-solubilisation PBS washes of the leaf material, which removed the bulk of the host contaminant proteins.

We obtained a purification recovery rate of LALF_32-51_-E7 of 30%. In comparison, recovery rates from *E*.*coli* were around 52% and they obtained yields of approximately 38 mg purified protein per liter of induced culture [44]. This relatively low recovery rate from plants can be attributed to the incomplete extraction of LALF_32-51_-E7 from leaf material and the manipulation of extracts during purification, and could almost certainly be improved upon. While the best conditions yet achieved for plant-produced LALF_32-51_-E7 were used, and it was expressed at levels up to 0.5% TSP or 50 mg/kg, this is lower than values reported for other E7-based plant-expressed proteins. For example, Buyel et al. (2012) [54] report the expression of a HPV-16 E7 fusion protein of 233 mg/kg FLW in *N*. *benthamiana*, and Whitehead et al. (2014) obtained expression levels of a Zera^®^-fused HPV-16 E7 protein of 150 mg/kg and levels of 1,100 mg/kg were obtained when the E7 protein sequence was shuffled (ZERA-16E7SH) [28]. This suggests that this protein might be detrimental to the plant cells. Since E7 can interact with other proteins besides the retinoblastoma protein [55,56], it is possible that it interferes with plant cell pathways, which is not seen for E7 expression in prokaryotic expression systems [42]. Furthermore, Demurtas *et al*., (2012) [52], reported a hexahistidine-tag being detrimental to the expression of transplastomic HPV-16 E7-based protein (E7GGG) in algae cells. When a different affinity tag was used, the protein expression level was increased.

The downstream processing of most recombinant proteins can account for 80% of the production cost, which in fact, currently represents a bottleneck in the development of low-cost biopharmaceuticals [24,40,57]. Therefore, it is important that an economic and efficient purification method is applied to increase the recovery of LALF_32-51_-E7. It could therefore be worth removing LALF_32-51_-E7’s His-tag and to test non-chromatographic purification strategies. For example, chloroplasts could be purified by differential ultra-centrifugation techniques and thereafter, LALF_32-51_-E7 could be extracted from the chloroplast envelopes [58,59]. Alternatively, targeting LALF_32-51_-E7 to secretory pathways and fusing it to carrier molecules could increase its expression levels, as demonstrated by Franconi et al. (2006) [60], for HPV-16 E7, and Massa et al. (2007) [61] and Buyel et al. (2012) [54] for E7GGG. Additionally, the HPV-16 E7 sequence used here could be replaced with the shuffled sequence used by Whitehead et al. (2014) [28], creating LALF_32-51_-16E7SH which could possibly be less toxic.

Overall, this study has demonstrated that the expression in a plant expression system of LALF_32-51_-E7, a recombinant protein with significant potential as a therapeutic for the treatment of HPV infections, is possible. We report here for the first time the expression, up-scaling of production, extraction and purification of plant-produced LALF_32-51_-E7. If further developed, this platform could potentially allow for the production of a low-cost and therefore accessible therapeutic vaccine for HPV-16. This would be extremely relevant in the context of developing countries, where cervical cancer and other HPV-related malignancies are most prevalent, and where the population has limited or no access to preventative vaccines.

## Materials and methods

### Bacterial strains and culture conditions

All constructs were maintained in DH5-α chemically competent *E*. *coli* cells (*E*. *cloni*^*™*^, Lucigen) grown in Luria-Bertani (LB) medium [1.0% tryptone, 0.5% yeast extract, 1.0% NaCl, pH 7.0 and 1.5% agar for solid medium]. Liquid cultures were grown with agitation at 120 revolutions per minute (rpm), at 37°C, for 16 to 18 h. Antibiotic selection was done using 100 μg/ml ampicillin.

*A*. *tumefaciens* strain GV3101 containing the helper plasmid pMP90RK, was used for PRIC3.0 and pTRAc constructs. *A*. *tumefaciens* strain LBA4404 was used for pEAQ-*HT* and pBIN constructs. Cells were grown in LB broth, with agitation at 120 rpm, at 27°C for 2–3 days. Antibiotic selection was done using 50 μg/ml carbenicillin (only used for recombinant GV3101::pMP90RK cells), 50 μg/ml rifampicin and 30 μg/ml kanamycin. To prevent clumping of LBA4404 cultures, liquid media were supplemented with MgSO_4_ to a final concentration of 2 mM.

### Construct generation and *Agrobacterium* electroporation

The E7 sequence in the LALF_32-51_-E7 fusion was previously modified to contain a base substitution (T/G) in the codon encoding for the first cysteine to abolish the carcinogenic effect of the protein. The LALF_32-51_ sequence encodes a small linear peptide containing residues 32 to 51 from the original LALF protein [17,18]. The LALF_32-51_-E7 sequence was codon-optimized for expression in *N*. *benthamiana*, synthesized by GenScript (USA). The LALF_32-51_-E7 sequence was inserted into the self-replicating plant expression vector pRIC3.0 using the RE sites Afl*III*/Xho*I*. The LALF_32-51_-E7 sequence was subcloned into thepRIC3.0-cTP vector using the Xho*I* and Mlu*I* restriction sites.

Electro-competent *A*. *tumefaciens* cultures were prepared as described in [62] and electroporated as described in [35]. GV3101::pMP90RK cells were electroporated with the constructs pTRAc-LALF_32-51_-E7, pRIC3.0 empty vector, pRIC3.0-LALF_32-51_-E7 and pRIC3.0-cTP-LALF_32-51_-E7. LBA4404 cells were electroporated with the constructs pEAQ-*HT* which contains the p19 expression cassette, pEAQ-*HT*-LALF_32-51_-E7 and pBIN-NSs.

### Agroinfiltration and transient expression of LALF_32-51_-E7 in *N*. *benthamiana leaves*

#### LALF_32-51_-E7 expression optimization in *N*. *benthamiana* leaves

Recombinant *A*. *tumefaciens* cultures were prepared for small scale agroinfiltration as described in [35]. Four to six-week-old *N*. *benthamiana* plants were used. The negative control, pRIC3.0 empty, was infiltrated at an OD_600_ of 1.0. Time trials were repeated three times independently, using an OD_600_ of 1.0, three plants per construct and vacuum infiltration. For vacuum infiltration, *A*. *tumefaciens* cultures were grown as described in [35] in LBB enriched medium [0.25% tryptone, 1.25% yeast extract, 0.50% NaCl, 10 mM 2-(N-Morpholino) ethanesulfonic acid (MES), pH 5.6], induced overnight with 20 μM acetosyringone and diluted to the desired final OD_600_ in resuspension solution [5 mM MES, 20 mM MgCl_2_, 0.2 mM acetosyringone].

#### Investigating the effect of silencing suppressors on the expression of LALF_32-51_-E7

Plants were vacuum infiltrated with pRIC3.0-LALF_32-51_-E7, pRIC3.0-cTP-LALF_32-51_-E7 and pRIC3.0 empty, with or without a silencing suppressor-containing construct: pEAQ-*HT*-P19 and pBIN-NSs. All *A*. *tumefaciens* cultures were set to an OD_600_ of 1.0. Three plants per construct were vacuum infiltrated. One leaf per plant was harvested on 3, 5 and 7 dpi, giving a total of 3 leaves per construct. This set of experiments was repeated twice, independently.

### Preparation of plant crude extracts

#### Small-scale expression studies

Vacuum-infiltrated plants, leaves were weighed immediately after being harvested. Leaves were flash-frozen in liquid nitrogen and ground up using a mortar and pestle. Crude extracts were prepared by homogenizing leaves in 2 v/w of extraction buffer [8 M urea in 1 mM carbonate-bicarbonate buffer, pH 10.6] using a T25 digital ULTRATTURRAX^®^ homogenizer (IKA). Homogenates were passed through a double-layer of Miracloth (EDM Chemicals) and were clarified twice by centrifugation at 13,000 rpm (BeckmanT Coulter Avanti^®^ J25TI centrifuge) for 10 min at room temperature.

#### Enhanced extraction strategy for LALF_32-51_-E7 purification studies

Vacuum-infiltrated leaves were flash-frozen in liquid nitrogen and ground up using a mortar and pestle. Ground leaf material was washed 3 times with phosphate buffered saline [PBS; 137 mM NaCl, 2.7 mM KCl, 10 mM PO_4_^3−^ (0.144% Na_2_HPO_4_ and 0.024% KH_2_PO_4_), pH 7.4] or 5–6 times with Triton-PBS [0.1%Triton X-100 (Sigma-Aldrich) in PBS buffer] by homogenizing in 4 v/w using a T25 digital ULTRATTURRAX^®^ homogenizer (IKA) and subsequently centrifuging at 13,000 rpm (Beckman T Coulter Avanti^®^ J25TI centrifuge) for 10 min at 4°C. LALF_32-51_-E7 was extracted from the washed and pelleted plant material with 2–5 v/w of urea extraction buffer at 4°C with shaking for 4 h. Extracts were passed through a double-layer of Miracloth and were clarified twice by centrifugation at 13,000 rpm for 20 min at room temperature. The final crude extracts were stored at -20°C.

### Purification of LALF_32-51_-E7 by Ni^2+^ affinity chromatography

LALF_32-51_-E7 crude extracts were prepared from 50 g FLW using the enhanced extraction strategy. Imidazole (Merck Millipore) and NaCl were added to a final concentration of 20 mM and 500 mM respectively. A 5 ml Ni^2+^ affinity column (HisTrap^™^ HP, GE Healthcare) was equilibrated with 5 column volumes (CV) of equilibration buffer [500 mM NaCl, 8 M urea, 20 mM imidazole in 50 mM Na-PO_4_ pH 8.0]. The crude extract was loaded onto the column using a 150 ml SuperLoop. Thereafter the column was washed with 10 CV of equilibration buffer or wash buffer [equilibration buffer at pH 6.5]. LALF_32-51_-E7 was eluted over 20 CV of a linear gradient (from 0% to 100%) of elution buffer [equilibration buffer with 500 mM imidazole] and an additional 5 CV step of 100% elution buffer. The ÄKTA Explorer^™^ system and the UNICORN 4.11 software (GE Healthcare) were used. All fractions were collected in 5 ml aliquots and stored at -20°C. Relevant fractions were analysed by western blots and SDS-PAGE gels.

Relevant elution fractions were pooled and extensively dialysed against 130 v/v renaturing buffer [10 mM Tris, pH 8.0]. The dialysis was carried over 4 h, followed by an overnight round and a final round of 4 h. Thereafter, dialysed samples were filter-sterilized through a 0.2 μm Corning^®^ syringe filters (Sigma-Aldrich) and stored at 4°C.

### Detection and quantification of LALF_32-51_-E7

#### Total soluble protein (TSP) and % TSP determination

Total soluble protein concentrations of crude extracts were determined using the DC Protein Assay (Bio-Rad). A serial dilution of 1 mg/ml to 0.06 mg/ml bovine serum albumin (BSA; Sigma-Aldrich) was used as standard. For %TSP determination, western blot densitometry was used. The plant-derived LALF_32-51_-E7 was quantified using the 6xHis Protein Ladder (QIAGEN^®^) as a protein standard. The anti-Histidine and goat anti-mouse IgG whole molecules conjugated to alkaline phosphatase (AP; Sigma-Aldrich) were used. The Syngene Gene Genius imaging system (Artisan Technology Group) and GeneTools software (Syngene) were used. The %TSP fold change was calculated using the equation bellow, where values above 1 indicated increase, a value of 1 indicated no change and a value below 1 indicated decrease.

$$Fold\;change\;=\frac{Final\;\%TSP\;value}{Initial\;\%TSP\;value}$$

### SDS-PAGE and western blotting

Crude extracts were mixed with 6x SDS sample application buffer [25% (v/v) glycerol, 0.5 M DDT, 5% (w/v) bromothymol blue] to a final concentration of 1x and boiled at 90°C for 10 min. Fifteen per cent SDS-PAGE-gels were loaded and electrophoresed in a Bio-Rad Tetra Cell system at 120 volts. Gels were stained with Aqua Stain^™^ (Vacutec) for 1 h, at 37°C, or with Coomassie Brilliant Blue stain (Bio-Rad) for 2 h at 37°C and destained with destaining solution [30% (v/v) methanol and 10% (v/v) glacial acetic acid in distilled water. Protein sizes were estimated using PageRuler^™^ Plus Prestained Protein Ladder (Thermo Scientific) or with the Colour Prestained Protein Standard, Broad Range (11–245 kDa) (NEB).

For western blots, proteins were transferred from SDS-PAGE-gels to nitrocellulose membranes using a Bio-Rad Trans-Blot^®^ Semi-dry transfer cell at 15 volts, for 1 h. The primary and secondary antibodies used were the polyclonal anti-HPV-16-E7 rabbit serum (I. Hitzeroth, Biopharming Research Unit, MCB, UCT) and the goat anti-Rabbit IgG whole molecules-AP (Sigma-Aldrich) unless otherwise stated. Blots were visualized with NBT/BCIP substrate (Sigma-Aldrich).

### Western blot densitometry

The plant-produced LALF_32-51_-E7 was quantified by western blot densitometry using a serial dilution of *E*. *coli*-produced LALF_32-51_-E7 inclusion bodies as a protein standard and anti-His antibodies as a detection method. The Syngene Gene Genius imaging system (Artisan Technology Group) and GeneTools software (Syngene) were used.

## Supporting information

S1 Fig Small-scale transient expression of LALF_32-51_-E7 in *N*. *benthamiana* leaves. (DOCX)
