# Supplementary material for: Expression optimization of a cell membrane-penetrating human papillomavirus type 16 therapeutic vaccine candidate in Nicotiana benthamiana
Source: PLoS One. 2017 Aug 11;12(8):e0183177. doi: 10.1371/journal.pone.0183177 (PMC5553638; doi:10.1371/journal.pone.0183177)
Supplement: S1 Fig — (DOCX) [file pone.0183177.s001.docx]

**Expression optimization of a cell membrane-penetrating Human papillomavirus type 16 therapeutic vaccine candidate in *Nicotiana benthamiana***

Short title: Expression optimization of LALF32-51-E7 in plants

Romana J. R. Yanez^1^, Renate Lamprecht^1^_,_ Milaid Granadillo^2^, Brandon Weber^3^, Isis Torrens^2^, Edward P. Rybicki^1, 4^ and Inga I. Hitzeroth^1*^

^1^Biopharming Research Unit, Department of Molecular and Cell Biology, University of Cape Town, Rondebosch, Cape Town, South Africa;

^2^Center for Genetic Engineering and Biotechnology, Cubanacan, Playa, Havana, Cuba;

^3^Structural Biology Research Unit, Division of Medical Biochemistry, Department of Clinical Laboratory Sciences, University of Cape Town, Observatory, Cape Town, South Africa;

^4^Institute of Infectious Disease and Molecular Medicine, University of Cape Town, Observatory, Cape Town, South Africa.

***Corresponding author**

E-mail: [inga.hitzeroth@uct.ac.za](mailto:inga.hitzeroth@uct.ac.za) (IIH)

# Supplementary data

## S1 Fig. Small-scale transient expression of LALF_32-51_-E7 in *N. benthamiana* leaves

To determine the best conditions that resulted in highest expression levels of LALF_32-51_-E7 for each construct, *N. benthamiana* leaves were syringe-infiltrated with recombinant *A. tumefaciens* cultures at different optical densities (OD_600_) and leaf discs were harvested over seven days. The pEAQ-*HT-*LALF_32-51_-E7 and pRIC3.0 empty construct were used as positive and negative controls, respectively.

Anti-E7 western blots showed that the expression of LALF_32-51_-E7 (≈22 kDa) using pRIC3.0 and pRIC3.0-cTP was successful (Fig S1). LALF_32-51_-E7 was absent or poorly detected for some pRIC3.0 samples (Fig S1, upper panel) but it was detected in all pRIC3.0-cTP samples (Fig S1, bottom panel). The expression of LALF_32-51_-E7 in pRIC3.0 appeared strongest at an OD_600_ between 1.0 and 1.5, on 3 days post infiltration (dpi). The expression in pRIC3.0-cTP appeared strongest at an OD_600_ between 0.5 and 1.0, also on 3 dpi. The expression of LALF_32-51_-E7 when using the positive control, pEAQ-*HT* was also detected, however, it appeared to be lower than that when using pRIC3.0 and pRIC3.0-cTP. No LALF_32-51_-E7 was detected in the negative control samples.


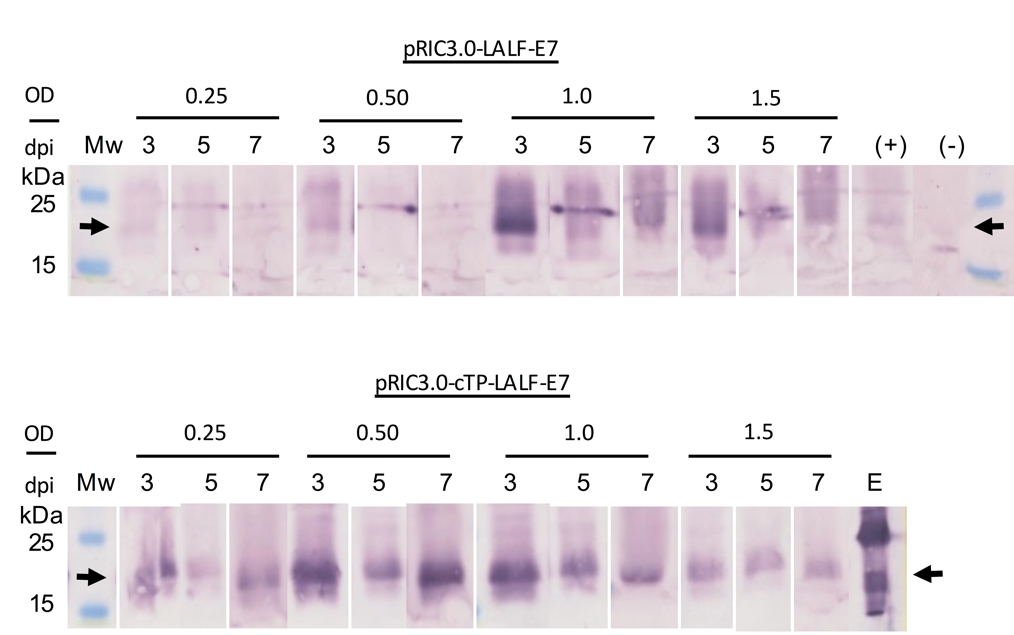


**Fig S1. Detection of LALF_32-51_-E7 small-scale expression in *N. benthamiana* leaves.** Western blots of equal volumes of crude extracts from *N. benthamiana* leaves syringe-infiltrated with pRIC3.0-LALF_32-51_-E7 (top panel) and pRIC3.0-cTP-LALF_32-51_-E7 (bottom panel). Different recombinant *A. tumefaciens* OD_600_s were used. Leaf clippings were harvested on 3, 5 and 7 dpi. (-), pRIC3.0 empty vector, harvested on 5 dpi. (+), pEAQ-*HT*-LALF_32-51_-E7, harvested on 5 dpi. E, purified *E. coli-*derived HPV-16 E7. Arrows indicate the expected position of LALF_32-51_-E7, ≈22 kDa. Each panel represents two nitrocellulose membranes. The images were re-arranged for aesthetic purposes. Antibody dilution of 1:1,000. Mw, molecular weight marker. dpi, days post infiltration.
